# Beyond Ergogenic Effects: Limited Evidence on Caffeine-Related Problematic Use, Dependence, and Withdrawal in Athletic Populations—A Structured Narrative Review

**DOI:** 10.3390/nu18172770

**Published:** 2026-08-25

**Authors:** Haowei Liu, Yang Cao, Guodong Zhang, Hansen Li

**Affiliations:** 1 School of Sports Training, Chengdu Sport University, Chengdu 641418, China; 2 Clinical Epidemiology and Biostatistics, School of Medical Sciences, Faculty of Medicine and Health, Örebro University, 70182 Orebro, Sweden; 3 Unit of Integrative Epidemiology, Institute of Environmental Medicine, Karolinska Institutet, 17177 Stockholm, Sweden; 4 International College, Krirk University, Bangkok 10220, Thailand; 5 School of Physical Education, Sichuan Agricultural University, Ya’an 625014, China

**Keywords:** caffeine, caffeine dependence, withdrawal, addiction, problematic caffeine use, athletes, sports nutrition

## Abstract

Caffeine is widely used in sport and exercise because of its established ergogenic effects, yet problematic use, dependence, and withdrawal in athletic populations remain poorly characterized. This structured narrative review used a systematic database search and prespecified selection process to identify studies directly addressing problematic caffeine use, dependence, use disorder, withdrawal, tolerance, habituation, or misuse in sport- and exercise-related populations. PubMed, Web of Science Core Collection, and Scopus were searched from inception to 15 June 2026. Four studies met the eligibility criteria: one cross-sectional study of caffeine dependence in university sport students, two experimental withdrawal studies, and one historical analysis of caffeine use and misuse in competitive cyclists. The evidence suggests that questionnaire-assessed dependence-related features, anxiety, self-reported withdrawal symptoms, withdrawal status, habitual intake, and performance may be interconnected; however, the studies were heterogeneous and generally did not use diagnostic assessments of caffeine use disorder. Athlete-specific prevalence and risk factors therefore remain unknown. Current evidence is insufficient for firm clinical or epidemiological conclusions, but it identifies an important gap between widespread ergogenic caffeine use and limited research on loss of control, continued use despite harm, withdrawal-driven use, and functional impairment. Standardized, athlete-specific research is urgently needed.

## 1. Introduction

Caffeine (1,3,7-trimethylxanthine) is a naturally occurring methylxanthine alkaloid and one of the most widely consumed pharmacologically active dietary components worldwide [1,2]. It is naturally found in plant sources such as coffee beans, tea leaves, cocoa beans, kola nuts, guarana, and yerba mate, and is also widely present in soft drinks, energy drinks, functional foods, over-the-counter medications, dietary supplements, and sports nutrition products [1,2]. Because caffeine promotes wakefulness, reduces fatigue, and enhances mental energy, its consumption has become deeply embedded in everyday life, study and work, and sports training. Coffee and other caffeine-containing products are widely consumed worldwide and have substantial global economic, social, and cultural relevance [3,4], while scientific production concerning coffee/caffeine and sport has increased markedly over the past two decades [5].

The main pharmacological actions of caffeine are generally attributed to its antagonism of adenosine receptors. By blocking adenosine-mediated inhibitory effects, caffeine can increase central nervous system excitability and influence alertness, attention, reaction speed, perceived fatigue, and functional performance under sleep-deprivation conditions [6,7]. In addition, caffeine may affect physical activity and exercise performance through multiple neuromuscular, metabolic, cardiovascular, and perceptual pathways [8,9]. Previous studies have shown that caffeine can improve various physical and cognitive outcomes, with potential ergogenic effects particularly evident in endurance exercise, maintenance of alertness during prolonged exercise, sport-specific endurance performance, and some high-intensity intermittent activities [7,10]. Therefore, in sport science and sports nutrition, caffeine is regarded not only as a common dietary component but also as a legal ergogenic substance supported by a relatively strong evidence base.

Caffeine has a long history of use in competitive sport and has long occupied an intersection between performance enhancement and anti-doping debates. Although caffeine was subsequently removed from the World Anti-Doping Agency Prohibited List, its use among athletes remains widespread. Based on 20,686 post-competition urine samples collected between 2004 and 2008, Del Coso et al. found that caffeine was detectable in most urine samples from elite athletes, indicating a high prevalence of caffeine use in competitive sport [11]. However, previous research on caffeine and sport has mainly focused on the effects of acute ingestion on exercise performance, the ergogenic effects of different doses and timing strategies, and the relationships of caffeine with training status, sport discipline, and habitual intake. By contrast, limited attention has been paid to how caffeine is used in athletic populations, and to whether problematic use, dependence, withdrawal, tolerance, craving, or excessive use may occur.

Athletes and physically active individuals are major users of caffeine. Many recreational athletes use highly concentrated caffeine-containing pre-workout supplements (commonly referred to in China as “nitrogen pump” products) to improve training performance, but this has also raised concerns about problematic use. As researchers and coaches, we have observed cases in practice in which recreational athletes consumed excessive caffeine before training, then had to rely on sedative medication at night to fall asleep, and refused to reduce the dose even when this behavior seriously affected their health and interpersonal relationships. These observations made us aware that caffeine misuse among exercisers may have certain addiction-like features, as other scholars have also cautioned [12]. Indeed, research on caffeine use disorder has suggested that caffeine-related problems should not be defined solely by high intake, but should instead consider features such as difficulty controlling use, continued use despite known adverse consequences, withdrawal symptoms, and functional impairment [13]. Studies on energy drinks also suggest that the rapid popularity of caffeinated products may lead to excessive intake, co-use with other substances, and insufficient recognition of health risks among young people [14]. Against this background, it is necessary to systematically summarize the sources, patterns, motives, measurement approaches, and main findings regarding caffeine and caffeinated product use among athletes and other sport- or training-related populations, and to further identify research gaps concerning problematic caffeine use, dependence, use disorder, withdrawal, and misuse risk.

Within the DSM-5-TR framework, caffeine intoxication and caffeine withdrawal are recognized disorders, whereas caffeine use disorder (CUD) remains a condition for further study rather than an established diagnosis. For the purposes of this review, we adopted a conservative CUD definition requires all three core features: a persistent desire or unsuccessful efforts to reduce or control use; continued use despite a persistent or recurrent physical or psychological problem caused or exacerbated by caffeine; and withdrawal or caffeine use to relieve or avoid withdrawal. Additional relavent indicators may include tolerance, craving, consuming more or for longer than intended, role or interpersonal impairment, and substantial time spent obtaining, using, or recovering from caffeine [13,15,16].

Prevalence estimates vary according to the diagnostic definition and sampling frame. A review of the earlier literature noted that 30% of 162 current caffeine users in a general-population survey met generic DSM-IV substance-dependence criteria when applied to caffeine, but no more than 9% met a conservative set approximating the three key DSM-5 CUD criteria [16]. In a later quota-based online sample of 1006 caffeine-consuming U.S. adults, 8% met all three proposed DSM-5 criteria. Meeting the criteria was associated with greater caffeine intake, younger age, cigarette smoking, poorer sleep, functional impairment, and higher depression, anxiety, and stress [17]. These findings indicate that clinically meaningful problematic use occurs in a minority of users and should not be equated with ordinary consumption.

At the population level, low-to-moderate caffeine consumption is generally well tolerated, but safety cannot be inferred from average intake alone. Evidence reviews identify dose, timing, individual sensitivity, age, pregnancy, comorbidity, and cumulative intake from multiple products as important modifiers of risk. Higher or late-day exposure may produce insomnia or reduced sleep, nervousness, jitteriness, anxiety, transient cardiovascular symptoms, and gastrointestinal discomfort, whereas abrupt cessation after regular use can cause headache, fatigue, irritability, and difficulty concentrating [1,18,19,20,21]. These concerns have particular relevance to sport because ergogenic use often involves concentrated pre-exercise doses, repeated use around training and competition, and simultaneous exposure from coffee, energy drinks, and multi-ingredient pre-workout products. Late-day dosing may compromise sleep and recovery, whereas habitual use and withdrawal may encourage re-dosing to avoid fatigue or headache. Nevertheless, the sport literature has primarily evaluated performance rather than loss of control, continued use despite harm, or functional impairment. This mismatch between widespread ergogenic use and sparse athlete-specific evidence on clinically meaningful problematic use defines the gap addressed by the present review.

## 2. Methods

### 2.1. Review Design and Rationale

This review was initially planned as a conventional systematic review. However, after systematic database searching and eligibility screening identified only four directly relevant studies, a conventional systematic synthesis or meta-analysis was neither feasible nor appropriate. We therefore reframed the work as a structured narrative review based on a systematic literature search, with an explicitly agenda-setting perspective. The search, screening, and data-extraction procedures remained prespecified and systematic, whereas the evidence synthesis was narrative and interpretive.

Accordingly, this review does not claim to provide pooled estimates or the depth expected from a mature systematic evidence base. Instead, it aims to map the sparse direct evidence, distinguish problematic caffeine use from ordinary or ergogenic use, and identify priorities for future research. Reporting of the search and study-selection procedures was informed by the PRISMA 2020 editorial overview [22], while the manuscript is presented as a Review rather than a Systematic Review.

### 2.2. Eligibility Criteria

Given that this review aimed to systematically summarize the main themes and core findings of research on problematic caffeine use, caffeine dependence, caffeine use disorder, tolerance, habituation, and withdrawal among sport and exercise populations, rather than evaluate a specific intervention effect, the eligibility criteria were developed with reference to both the PICOS and PECO frameworks. The PICOS framework was used primarily to structure eligibility criteria for experimental studies [23], whereas the PECO framework was used primarily to structure eligibility criteria for observational exposure studies [24].

The operationalized PICOS and PECO criteria are presented in Table 1 and Table 2.

Eligible populations included athletes, collegiate or student athletes, recreational athletes, regular exercisers, fitness participants, resistance- or endurance-training participants, physically active adults, and other groups explicitly studied in sport, training, or fitness contexts. General-population studies were included only when sport- or exercise-related subgroup data were reported or when caffeine use was examined in an exercise context.

Eligible studies had to involve caffeine or caffeinated products, including coffee, tea, energy drinks, caffeine capsules or tablets, caffeine gels, chewing gum, pre-workout supplements, multi-ingredient supplements, or other caffeine sources. Studies focused solely on acute ergogenic effects were included only when they also addressed habitual use, tolerance, habituation, withdrawal, dependence, problematic use, adverse effects, or safety.

Eligible comparators included placebo, caffeine-free or low-dose conditions, different caffeine doses, habitual versus non-habitual users, ingestion versus withdrawal conditions, pre-post comparisons, or no comparator when relevant information was provided. Outcomes included problematic caffeine use, dependence, use disorder, addiction, withdrawal symptoms, tolerance, habituation, craving, misuse, difficulty reducing intake, excessive use, sleep problems, anxiety, mood changes, fatigue, headache, reduced attention, adverse reactions, or other caffeine-related psychological, behavioral, physiological, or health outcomes. No restrictions were placed on study design, provided the record was an original research article.

### 2.3. Exclusion Criteria

The following records were excluded:

1. Non-English-language studies;
2. Animal or in vitro studies;
3. Studies that did not involve sport, exercise, fitness, or physically active populations;
4. Studies that examined only the acute effects of caffeine on exercise performance without addressing habitual use, tolerance, habituation, withdrawal, dependence, problematic use, or adverse patterns of use;
5. Studies that reported only general-population results and from which sport- or exercise-related population information could not be extracted;
6. Studies that did not involve caffeine or caffeinated products;
7. Studies that did not report any information related to problematic use, dependence, use disorder, withdrawal, tolerance, habituation, psychological or behavioral outcomes, or health risks;
8. Conference abstracts, editorials, commentaries, opinion articles, news reports, or articles without original data, unless they were used only for background discussion or citation tracking;
9. Studies for which the full text was unavailable or the full-text information was insufficient for data extraction.

### 2.4. Information Sources

To provide complementary biomedical and multidisciplinary coverage, this study searched the following three major bibliographic databases:

1. PubMed;
2. Web of Science (all databases and all collections);
3. Scopus.

The search period covered each database from inception to 15 June 2026. In addition to database searches, the reference lists of included studies and relevant reviews were manually checked to identify potentially missed studies. For key publications in this field, citation tracking was also conducted through Google Scholar to supplement the evidence base.

### 2.5. Search Strategy

The search strategy was structured around three core concept blocks: caffeine exposure, concepts related to problematic use/dependence/withdrawal, and sport and exercise populations. Keyword selection and search-term design were informed by relevant reviews and bibliometric studies [25]. The core Boolean search strategy is presented in Supplementary Table S1. In PubMed, the title and abstract fields were searched; in Web of Science, topic searches were used; and in Scopus, the title, abstract, and keyword fields were searched. The complete database-specific Boolean strings and field tags are reported in Supplementary Table S1.

### 2.6. Reference Management and Study Selection

All database search results were imported into EndNote (version 8.0) for management, and duplicates were removed before screening. Study selection was conducted in two stages. In the first stage, titles and abstracts were screened to exclude records that clearly did not meet the eligibility criteria. In the second stage, the full texts of potentially relevant records were obtained and assessed according to the inclusion and exclusion criteria.

Study selection was completed independently by two reviewers. Disagreements were resolved through discussion; if consensus could not be reached, a third reviewer adjudicated. Reasons for full-text exclusion were recorded and classified into the following categories: ineligible population, ineligible research concept, ineligible context, no relevant caffeine-related outcome, focus only on acute exercise performance, non-English-language publication, insufficient information, or unavailable full text.

### 2.7. Data Extraction

A prespecified standardized data-extraction form was used to extract information from the included studies. Extracted information included:

1. Basic bibliographic information: authors, publication year, and country or region;
2. Study design: cross-sectional study, experimental study, cohort study, qualitative study, mixed-methods study, and so on;
3. Sample characteristics: sample size, age, sex, sport discipline, training status, and competitive level;
4. Population category: elite athletes, collegiate athletes, recreational exercisers, fitness participants, physically active adults, and so on;
5. Caffeine source: coffee, energy drinks, caffeine capsules, caffeine chewing gum, caffeine gels, pre-workout supplements, multi-ingredient pre-workout supplements, and so on;
6. Caffeine exposure characteristics: dose, frequency, timing, habitual intake status, duration of use, and so on;
7. Caffeine-related concepts investigated: problematic use, dependence, use disorder, withdrawal, tolerance, habituation, craving, misuse, or excessive use;
8. Measurement tools or assessment methods;
9. Main outcome measures;
10. Main findings.

Data extraction was performed by one reviewer and checked by another reviewer.

### 2.8. Evidence Synthesis

Given the limited number of included studies and the anticipated heterogeneity in study design, population type, caffeine source, measurement tools, and outcome measures, no meta-analysis was planned. All results were integrated using a narrative synthesis approach.

## 3. Results

### 3.1. Study Selection Process

**Figure 1:**
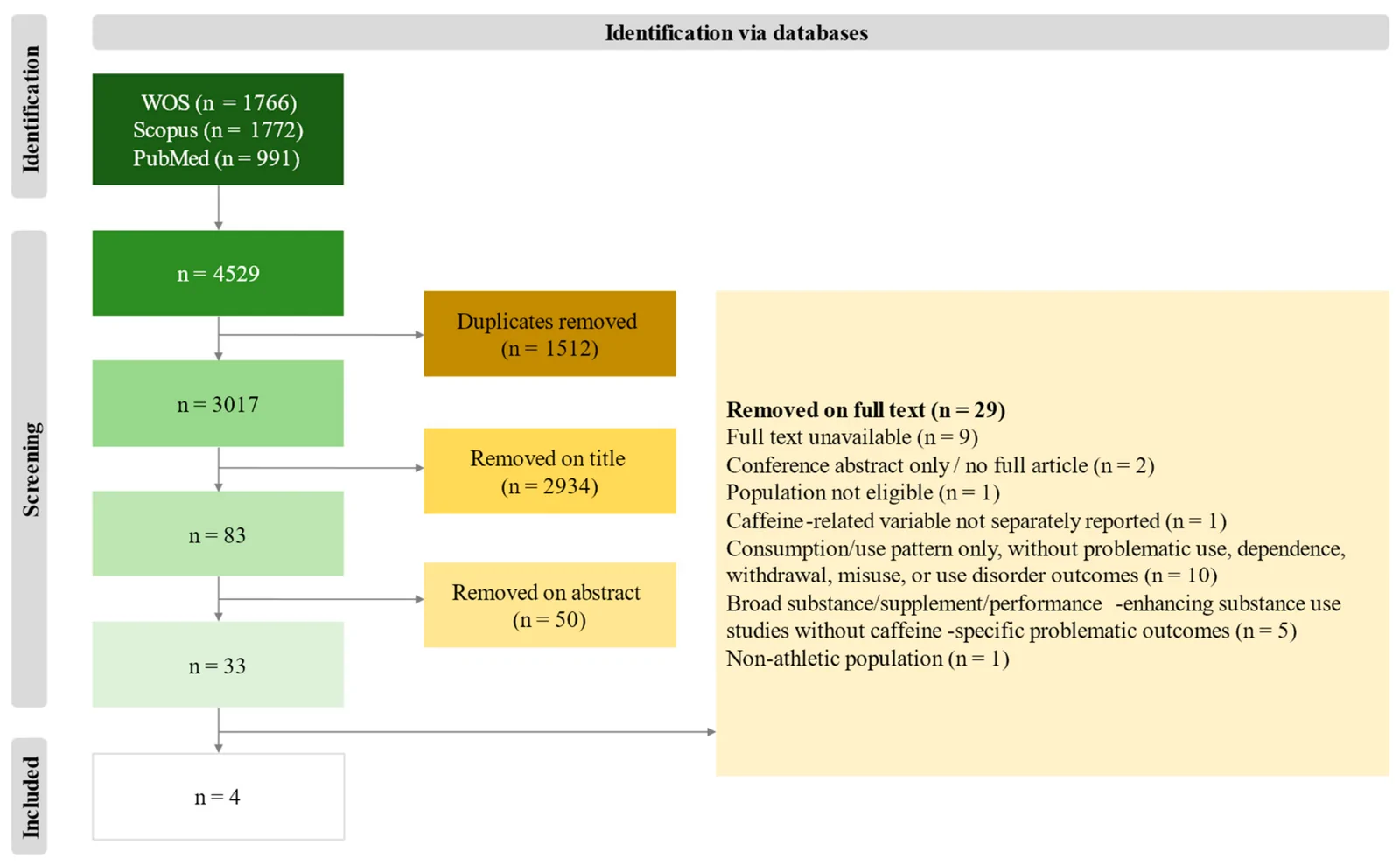
Flow diagram of the study-selection process.

We identified a total of 4529 records from three major databases. After excluding obviously irrelevant studies based on their titles and abstracts, we conducted a more detailed screening and further excluded articles that did not explicitly focus on athletes, lacked substantial research content, or addressed problematic or harmful caffeine use. Notably, studies with unclear reporting or inaccessible full texts were also excluded. Ultimately, only four articles were included, as they were considered relevant to the topic (Figure 1). No additional evidence was identified through citation tracking using Google Scholar.

### 3.2. General Characteristics of the Included Studies

Four studies were ultimately included, all of which directly addressed issues related to caffeine dependence, withdrawal, or misuse among athletic populations. The included studies were published between 1984 and 2026, indicating that this topic has existed for a long period but has not developed into a continuous and systematic body of research. Study designs included cross-sectional correlational research, placebo-controlled repeated-measures and crossover experiments incorporating double-blind caffeine-placebo comparisons, and observational analyses based on doping-control urine samples. Participants included university sport students, well-trained endurance athletes, recreational athletes, and competitive cyclists. Overall, the direct evidence is very limited, and research questions and measurement approaches are scattered. The available evidence is therefore insufficient to draw firm conclusions about the prevalence of problematic caffeine use, caffeine dependence, or caffeine use disorder among athletic populations. Detailed study characteristics are presented in Table 3.

### 3.3. Evidence Related to Caffeine Dependence and Use Disorder

Among the final included studies, Singh et al. [26] provided the most direct investigation of questionnaire-assessed caffeine dependence/use-disorder features in an athletic population. The study focused on university sport students, used the Caffeine Use Disorder Questionnaire (CUDQ) to assess dependence/use-disorder features, and examined anxiety as a statistical mediator of the association between CUDQ scores and reaction time during a Stroop task. Its value lies in moving beyond intake frequency or product sources and placing caffeine-related problems within a framework of questionnaire-assessed dependence-related features, anxiety, and cognitive performance. It therefore provides comparatively direct, although not diagnostic, evidence of possible problematic caffeine use among sport students.

Nevertheless, this study has clear limitations. First, its cross-sectional design cannot determine the causal direction among CUDQ scores, anxiety, and reaction time during the Stroop task. Second, the participants were university sport students, and the findings cannot be directly generalized to professional athletes, adolescent athletes, endurance athletes, or fitness populations. Third, although the study used a tool related to caffeine use disorder, the applicability, diagnostic thresholds, and cross-cultural stability of this tool in athletic populations still require further validation. Therefore, current evidence regarding caffeine dependence/use disorder among athletic populations remains at a very early stage.

### 3.4. Caffeine Withdrawal, Habitual Intake, and Exercise Performance

Two experimental studies directly involved controlled caffeine withdrawal. Irwin et al. [27] examined high-intensity endurance cycling performance in well-trained male cyclists and triathletes under caffeine-withdrawal and acute caffeine-ingestion conditions. Van Soeren and Graham [28] examined metabolic, endurance, and catecholamine responses after caffeine ingestion among habitual high caffeine consumers following zero, two, or four days of withdrawal. Together, these studies show that withdrawal status and habitual intake are relevant to the interpretation of caffeine’s exercise effects, while also demonstrating that acute caffeine can improve performance under both withdrawal and non-withdrawal conditions.

However, the primary aims of these studies were to investigate caffeine-related performance or physiological effects rather than to conduct standardized diagnostic assessments of caffeine withdrawal or caffeine use disorder. Both studies collected non-standardized subjective reports of withdrawal symptoms. In the 12-participant cycling study, 11 participants reported at least one symptom, including headache (*n* = 9), fatigue (*n* = 8), and reduced focus or motivation (*n* = 5). In the six-participant study, all participants reported symptoms lasting 2–4 days, including severe headache, fatigue, lethargy, and flu-like symptoms. Nevertheless, neither study used a validated standardized withdrawal-symptom scale, assessed craving or difficulty controlling use, evaluated functional impairment, or performed a diagnostic assessment. These studies therefore provide direct evidence of subjective withdrawal-related symptoms and performance or physiological phenomena in athletic populations, but they are not epidemiological studies of caffeine-withdrawal disorder or caffeine use disorder and cannot establish prevalence or clinical severity.

### 3.5. Early Evidence on Caffeine Use and Misuse in Sport

Delbeke and Debackere [29] provided early evidence on caffeine use and misuse in sport. Using doping-control urine samples from competitive cyclists, they compared urinary caffeine concentrations across competitive categories and discussed a potential competition threshold. The study is historically important because it shows that caffeine occupied an intersection between performance enhancement, anti-doping regulation, and potential misuse long before contemporary energy drinks and pre-workout supplements became common.

However, the historical context of this study differs markedly from the contemporary sports-supplement environment. Modern athletes have more diverse caffeine sources, including energy drinks, pre-workout supplements, caffeine gels, chewing gum, and multi-ingredient supplements, whereas early doping-control studies mainly reflected urinary caffeine levels among competitive cyclists at a specific time. In addition, although urinary caffeine concentration can reflect exposure level, it cannot directly assess dependence, withdrawal, craving, or difficulty controlling use. Therefore, this study is better interpreted as historical evidence of caffeine “use and misuse” rather than direct diagnostic evidence of current caffeine use disorder.

### 3.6. Evidence Synthesis and Research Gaps

Taken together, these four studies show that research on problematic caffeine use, dependence, withdrawal, and misuse among athletic populations is extremely limited. The available evidence comes from a dependence/use-disorder-related survey among university sport students, withdrawal experiments among endurance athletes or habitual users, and an early discussion of misuse based on doping-control urine samples from competitive cyclists. Collectively, these studies suggest that caffeine in athletic populations is not only an ordinary dietary component or ergogenic supplement, but may also involve issues such as dependence, withdrawal, excessive use, or misuse. However, the current evidence cannot answer several key questions, such as the true prevalence of caffeine use disorder among athletic populations, which sport disciplines or populations are at higher risk, whether caffeine-withdrawal symptoms affect training and competition, and whether energy drinks and pre-workout supplements increase the risk of problematic use.

Therefore, the core finding of this review is not that caffeine use disorder among athletic populations has been sufficiently established, but rather that, despite the widespread use of caffeine in sport, very few studies have directly examined its problematic use, dependence, and withdrawal. This evidence gap indicates that future research should use standardized tools for assessing caffeine use disorder or dependence, conduct cross-sectional, longitudinal, and experimental studies across different sport disciplines, competitive levels, and age groups, and integrate indicators such as caffeine intake, withdrawal symptoms, craving, sleep, anxiety, training load, and exercise performance. Such work is needed to more comprehensively understand the forms and potential consequences of caffeine-related problematic use among athletic populations.

## 4. Discussion

### 4.1. Main Findings

This review focused on evidence related to problematic caffeine use, dependence, withdrawal, misuse, and related risks among athletic populations. After full-text screening and thematic reassessment, only four studies directly met the topic of this review. The included studies addressed features related to caffeine dependence/use disorder among university sport students, withdrawal-related experiments in well-trained or habitual caffeine-using athletic populations, and early doping-control evidence of caffeine use and misuse among competitive cyclists. Overall, the direct evidence is very limited, and study designs, sample sources, measurement approaches, and research questions are scattered. Therefore, the most important finding of this review is not that caffeine use disorder among athletic populations has been fully demonstrated, but that, despite extensive research and use of caffeine in sport, studies that truly focus on problematic use, dependence, withdrawal, or misuse remain extremely scarce.

The included performance studies can be interpreted against caffeine’s established ergogenic mechanisms. By antagonizing central adenosine A1 and A2A receptors, caffeine can increase arousal and neural drive while reducing perceived effort and central fatigue; peripheral effects may also contribute depending on dose and task [6,7]. Irwin et al. [27] found that 3 mg/kg improved cycling time-trial performance both after withdrawal and during the controlled low-dose caffeine condition, while Van Soeren and Graham [28] observed longer time to exhaustion across caffeine trials regardless of withdrawal duration and concluded that the benefit was not explained by the measured hormonal or metabolic responses. Together, these findings argue against a purely withdrawal-reversal explanation and suggest that the measured hormonal and metabolic responses do not fully account for the ergogenic effect; however, neither study established the underlying central or peripheral mechanism. Conversely, cross-sectional associations of higher CUDQ scores with greater anxiety and slower reaction time during a Stroop task were reported in sport students [26]. Because these variables were measured contemporaneously, their temporal and causal directions cannot be determined.

### 4.2. Comparison with General-Population Evidence and Conceptual Boundaries

An important methodological judgment in this review is that general caffeine use, energy drink consumption, or sports supplement use is not equivalent to problematic caffeine use. Many studies in athletic populations have reported the prevalence, sources, motives, and knowledge levels related to caffeine, energy drinks, or caffeine-containing supplements. These studies are valuable for demonstrating that caffeine use is common, sources are diverse, and performance-enhancement motives exist among athletic populations. However, if they do not address dependence, withdrawal, tolerance, craving, difficulty controlling use, misuse, or continued use despite adverse consequences, they cannot serve as core evidence for caffeine use disorder or problematic use.

This distinction is important for interpreting the present review. Athletes’ use of caffeine does not necessarily indicate a problem; caffeine intake at appropriate doses and timing can be part of a sports nutrition strategy. The International Olympic Committee consensus statement on dietary supplements for high-performance athletes also notes that some supplements, including caffeine, may provide performance benefits in specific contexts, but supplement use should still be judged according to safety, legality, and individualized needs [30]. The key concern arises when use becomes compulsive, uncontrolled, withdrawal-driven, or functionally impairing, at which point caffeine may shift from ordinary supplement use to problematic use. Future studies should therefore not only ask “whether athletes use caffeine”, but should further assess “whether athletes have difficulty controlling use”, “whether discomfort occurs after stopping use”, “whether use continues to avoid withdrawal”, and “whether athletes continue use despite known effects on sleep, anxiety, or health”.

Compared with general-population evidence, athlete-specific prevalence estimates are not yet available. In the U.S. adult sample reported by Sweeney et al. [17], 8% met all three proposed CUD criteria, whereas the sport-student study reported associations among questionnaire scores, anxiety, and reaction time during a Stroop task but did not provide a prevalence estimate based on a validated diagnostic interview. It is therefore not possible to determine whether athletes have a higher prevalence than non-sport populations. However, the correlates identified in general adults—greater caffeine intake, younger age, smoking, poorer sleep, anxiety, depression, stress, and functional impairment—are directly relevant to athlete screening and should be measured alongside sport-specific exposures [17].

General-population evidence also clarifies why high intake alone should not be labeled CUD. The clinically meaningful boundary is loss of control, withdrawal-driven use, continued use despite harm, or impairment, not the mere presence of daily caffeine consumption. This distinction supports the present review’s decision to exclude studies that reported only prevalence or motives for ordinary caffeine use without dependence-, withdrawal-, misuse-, or impairment-related outcomes.

### 4.3. Energy Drinks, Pre-Workout Supplements, and Potential Risks

Although the number of core included studies was limited, energy drinks and pre-workout supplements remain important sources of caffeine-related risk among athletic populations when viewed from the broader sports-nutrition context. Energy drinks and multi-ingredient pre-workout supplements often contain not only caffeine but also sugar, taurine, guarana, beta-alanine, creatine, citrulline, or other stimulant ingredients. Compared with coffee or standardized caffeine capsules, these products are more likely to be driven by commercial marketing, peer influence, and social media promotion, and may also make users more likely to underestimate their actual caffeine intake.

Previous literature suggests that the safety of energy drinks and caffeinated products deserves attention. Early work noted that the popularity of caffeinated energy drinks may lead to excessive intake and co-use with other substances [14]. The American Academy of Pediatrics also clearly distinguished sports drinks from energy drinks and noted that energy drinks contain caffeine and other stimulant ingredients and are not appropriate for routine use by children and adolescents [31]. A systematic review and meta-analysis further showed that energy drink consumption was associated with increased risks of adverse events such as insomnia, jitteriness, or restlessness [32]. In addition, caffeine safety reviews and the EFSA safety opinion indicate that caffeine dose, individual sensitivity, timing of intake, and cumulative intake from multiple sources may all influence safety [18,20]. Therefore, even though studies on energy drinks or pre-workout supplements were not included in the core analysis, they provide important background for understanding potential problematic caffeine use among athletic populations.

Adverse-effect evidence provides a second bridge between the included studies and the broader literature. The anxiety association reported by Singh et al. [26] is consistent with dose- and sensitivity-dependent anxiogenic responses; the withdrawal manipulations used by Irwin et al. [27] and Van Soeren and Graham [28] generated non-standardized reports of symptoms such as headache, fatigue, lethargy, reduced concentration or motivation, and flu-like symptoms, broadly consistent with the established withdrawal syndrome; and the historical urinary concentrations reported by Delbeke and Debackere [29] indicate exposure but not a clinical disorder. Sleep is especially relevant because late caffeine intake can delay or fragment sleep, impaired recovery may increase the perceived need for caffeine, and repeated use to counter sleep loss may create a self-reinforcing cycle [19,20,21]. These parallels support balanced monitoring of performance benefits together with sleep, anxiety, withdrawal, and functional consequences.

### 4.4. Limitations of the Included Evidence

The included studies have clear limitations. First, the number of studies is extremely small, and their publication years span a long period, indicating a lack of continuous research accumulation in this field. Second, the study designs are highly heterogeneous, including cross-sectional research, crossover experiments, and analyses of doping-control samples, making direct comparisons difficult. Third, except for the study by Singh et al. [26], the remaining studies did not use standardized caffeine use disorder or dependence assessment tools. The withdrawal-related studies mainly focused on exercise performance and physiological responses and recorded symptoms only through non-standardized subjective reports; they did not use validated withdrawal-symptom scales or assess craving, difficulty controlling use, or functional impairment. Fourth, the samples were limited and mainly involved university sport students, endurance athletes, recreational exercisers, and cyclists, and therefore cannot represent all athletic populations. Fifth, modern caffeine sources have expanded from traditional coffee to energy drinks, pre-workout supplements, caffeine gels, chewing gum, and multi-ingredient sports supplements, but the existing core studies have not adequately captured these newer exposure sources.

Therefore, the current evidence is insufficient to support strong conclusions about the prevalence, risk factors, or health consequences of caffeine use disorder among athletic populations. A more accurate interpretation is that the small body of available evidence suggests that this issue warrants attention, but the field remains at an early stage.

### 4.5. Limitations of the Review Process

Although this review followed the logic of systematic searching and screening, it still has process-level limitations. First, terminology related to problematic caffeine use is inconsistent across the literature and may include dependence, use disorder, withdrawal, abuse, misuse, problematic use, substance use, or performance-enhancing substance use, which increased the difficulty of searching and screening. Second, under the strict eligibility criteria of this review, studies that only described patterns of caffeine consumption, energy drink use, or supplement use were excluded. This approach improved topical focus but also means that this article does not provide a comprehensive review of the overall prevalence of caffeine use among athletic populations. Third, studies for which full texts could not be obtained were excluded, which may have introduced full-text availability bias. Fourth, because only four core studies were ultimately included, this review was not suitable for meta-analysis and cannot provide pooled quantitative estimates. Because the manuscript was reframed after screening revealed an unexpectedly sparse evidence base, the synthesis includes an explicitly interpretive and agenda-setting component and should not be read as a conventional systematic review or as a basis for pooled effect estimates.

### 4.6. Practical Implications

Despite the limited evidence, this review has practical implications. First, sports nutrition practice should avoid treating caffeine simply as a “safe and effective” ergogenic tool. The effectiveness of caffeine does not mean that there is no risk of problematic use. For athletes who frequently use caffeine, energy drinks, or pre-workout supplements, coaches, sports nutritionists, and sports medicine practitioners should consider total intake, timing of intake, sleep quality, anxiety responses, palpitations, gastrointestinal discomfort, and signs of difficulty reducing use. The effects of caffeine intake on sleep and recovery are particularly important, because sleep insufficiency may itself encourage athletes to rely further on caffeine, thereby forming a potential cyclical pattern of use.

Second, athlete education should distinguish between “appropriate use” and “dependence-like use”. Appropriate use emphasizes dose, timing, and individualized responses, whereas problematic use involves difficulty controlling intake, withdrawal discomfort, excessive reliance, and continued use despite negative consequences. In adolescent athletes, university sport students, endurance athletes, and high-frequency fitness participants in particular, the use of energy drinks and pre-workout supplements may increase caffeine exposure from multiple sources and therefore requires targeted education.

Third, caffeine can serve as an entry point for education on sports-supplement risk. It is not a prohibited substance, but its legality, availability, and ergogenic effects may cause athletes to underestimate potential risks. Through this common substance, athletes can be guided to understand supplement use, dose control, product-label interpretation, cumulative intake from multiple sources, and the risk of behavioral dependence.

### 4.7. Future Research Directions

Future research should proceed in several directions. First, epidemiological studies using standardized tools should be conducted in different athletic populations to assess the occurrence of caffeine dependence, caffeine use disorder, problematic use, withdrawal, tolerance, and craving. Second, caffeine exposure should be measured more precisely by recording total intake from coffee, tea, energy drinks, pre-workout supplements, caffeine tablets, capsules, gels, chewing gum, and multi-ingredient supplements. Third, longitudinal studies should examine whether caffeine use changes with training age, competitive level, competition pressure, sleep insufficiency, and training load. Fourth, experimental studies should measure both exercise performance and withdrawal symptoms with validated standardized instruments, including fatigue, headache, mood changes, reduced attention, craving, and changes in training motivation. Fifth, particular attention should be paid to high-risk populations, such as adolescent athletes, university sport students, endurance and ultra-endurance athletes, high-frequency gym users, and multiple-supplement users.

In addition, future research should clearly distinguish among ordinary use, functional use, high-risk use, misuse, abuse, dependence, and use disorder. Only when concepts and measurement tools become clearer will it be possible to determine the scale and clinical significance of caffeine-related problematic use among athletic populations.

## 5. Conclusions

This review identified only four studies that directly addressed caffeine dependence, withdrawal, or misuse in sport and exercise populations. Their heterogeneous designs and limited measurement of loss of control, continued use despite harm, and functional impairment preclude estimates of athlete CUD prevalence or risk. Caffeine should therefore be interpreted as an evidence-based ergogenic aid whose benefits can coexist with sleep, anxiety, withdrawal, and problematic-use concerns in susceptible athletes.

## Figures and Tables

**Table 1:** PICOS framework used for experimental studies.

Framework Item	Operational Definition
Population	Athletes, collegiate or student athletes, recreational athletes, regular exercisers, fitness participants, resistance- or endurance-trained participants, physically active adults, and other sport-, training-, or fitness-related populations.
Intervention/exposure	Caffeine or caffeinated products, including coffee, tea, energy drinks, capsules/tablets, gels, chewing gum, pre-workout supplements, and multi-ingredient supplements; acute ingestion, habitual use, or controlled withdrawal.
Comparator	Placebo, caffeine-free or low-dose conditions, different doses, habitual versus non-habitual users, withdrawal versus maintenance conditions, or pre-post comparisons.
Outcomes	Problematic use, dependence, use disorder, withdrawal, tolerance, habituation, craving, misuse, difficulty reducing intake, excessive use, sleep, anxiety, mood, cognition, adverse reactions, physiological responses, or other health outcomes.
Study design	Original experimental research, including randomized, placebo-controlled, crossover, or other intervention designs.

**Table 2:** PECO framework used for observational studies.

Framework Item	Operational Definition
Population	Athletes, collegiate or student athletes, recreational athletes, regular exercisers, fitness participants, resistance- or endurance-trained participants, physically active adults, and other sport-, training-, or fitness-related populations.
Exposure	Caffeine or caffeinated-product exposure characterized by source, dose, frequency, timing, habitual-use status, duration, urinary concentration, withdrawal, or related use pattern.
Comparator	Non-users or lower-exposure groups, different use-pattern or competitive categories, exposed versus unexposed groups, or no comparator when the study provided relevant descriptive evidence.
Outcomes	Problematic use, dependence, use disorder, withdrawal, tolerance, habituation, craving, misuse, difficulty reducing intake, excessive use, sleep, anxiety, mood, cognition, adverse reactions, physiological responses, or other health outcomes.
Study design	Original observational research, including cross-sectional, cohort, case–control, correlational, qualitative, mixed-methods, or laboratory surveillance studies.

**Table 3:** Characteristics and main findings of the included studies.

Author (Year)	Country/Region	Study Design	Sample Characteristics	Population Category	Caffeine Source/Exposure Characteristics	Measurement Tools or Assessment Methods	Main Findings
[26]	India	Cross-sectional study; mediation analysis	*n* = 300 university sport students; 158 men and 142 women; aged 18–25 years; five private universities in Punjab; all trained or were physically active for at least 2 h/day.	University sport students	Caffeine sources were not quantified. The background mentioned coffee, tea, soft drinks, energy drinks, and pre-workout blends. Dose, frequency, and duration were not reported.	Caffeine Use Disorder Questionnaire (10 items; DSM-5-based); Hamilton Anxiety Rating Scale; PsyToolkit Stroop test; Pearson correlations and mediation analysis.	Higher CUDQ scores correlated with anxiety (r = 0.47) and slower Stroop reaction time (r = 0.43); anxiety also correlated with slower Stroop reaction time (r = 0.54). Anxiety showed partial statistical mediation in this cross-sectional model; causality cannot be inferred.
[27]	Australia	Randomized double-blind placebo-controlled crossover trial	*n* = 12 well-trained male cyclists and triathletes; age 28.3 ± 5.8 years; VO2peak 63.7 ± 7.4 mL·kg^−1^·min^−1^; habitual caffeine intake 240 ± 162 mg/day (range 18–469).	Well-trained male endurance athletes (cyclists and triathletes)	Four-day dietary caffeine abstinence; 1.5 mg/kg/day caffeine or placebo on days 1–4; acute 3 mg/kg caffeine or placebo 90 min before exercise on day 5.	Four randomized double-blind crossover conditions; 1-h cycling time trial at 75% peak sustainable power; plasma caffeine and post-trial symptom/treatment-identification questionnaires.	Acute caffeine improved performance by 3.0% after withdrawal and 3.6% without withdrawal; withdrawal did not enhance the ergogenic effect. Symptoms were reported by 11/12 participants, chiefly headache (*n* = 9), fatigue (*n* = 8), and reduced focus/motivation (*n* = 5).
[28]	Canada	Placebo-controlled repeated-measures experiment with randomized double-blind caffeine-placebo comparisons; final placebo repeat was single-blind	*n* = 6 male recreational athletes; age 36.7 ± 4.2 years; VO2max 54.5 ± 3.3 mL·kg^−1^·min^−1^; habitual intake 761.3 ± 11.8 mg/day (means ± SE), mainly from coffee.	Recreational athletes/habitual high caffeine consumers	Compared no withdrawal, 2-day withdrawal, and 4-day withdrawal; 6 mg/kg caffeine capsules or dextrose placebo 1 h before cycling to exhaustion at 80–85% VO2max.	Seven trials at least 10 days apart; randomized double-blind caffeine-placebo dosing within phases and a single-blind final placebo repeat; plasma caffeine, subjective symptoms, metabolic and hormonal measures.	Caffeine prolonged time to exhaustion regardless of withdrawal duration. All six participants reported 2–4 days of symptoms, including severe headache, fatigue, lethargy, and flu-like symptoms. The ergogenic effect was not fully explained by measured metabolic or hormonal responses.
[29]	Belgium	Doping-control urine-sample analysis/observational laboratory study	Controls were compared with 775 cyclists tested during the 1982 season; categories included professional, amateur, beginner, and junior cyclists. Several coffee drinkers also underwent 60-h urinary caffeine monitoring.	Competitive cyclists; doping-control samples	Urinary caffeine concentration; specific sources and doses were not stated in the available abstract; 60-h urinary monitoring in several coffee drinkers.	Urinary caffeine measurement; comparison of controls and doping-control samples; analysis across cycling categories.	Caffeine (mis)use was more evident in professional and amateur cyclists than in beginner and junior cyclists. The authors proposed a urinary caffeine maximum level of 15 μg/mL in competition.

## Data Availability

No new data were created or analyzed in this study. Data sharing is not applicable to this article.

## Supplementary Materials

The following supporting information can be downloaded at: https://www.mdpi.com/article/10.3390/nu18172770/s1, Supplementary Table S1. Database-specific search strategies used from database inception to 15 June 2026.
