# Supplementary material for: Beyond Ergogenic Effects: Limited Evidence on Caffeine-Related Problematic Use, Dependence, and Withdrawal in Athletic Populations—A Structured Narrative Review
Source: Nutrients. 2026 Aug 25;18(17):2770. doi: 10.3390/nu18172770 (PMC13567338; doi:10.3390/nu18172770)
Supplement: Supplementary file 1 [file nutrients-18-02770-s001.zip › nutrients-4461057-supplementary.pdf]

Supplementary Materials

Supplementary Table S1. Database-specific search strategies used from database inception to 15 June 2026.

Database	Search field and complete Boolean query	Search date
PubMed	(caffeine[Title/Abstract] OR caffeinated[Title/Abstract] OR "caffeine supplement*"[Title/Abstract] OR "caffeine supplementation"[Title/Abstract] OR "caffeine intake"[Title/Abstract] OR "caffeine consumption"[Title/Abstract] OR "caffeine use"[Title/Abstract] OR coffee[Title/Abstract] OR "energy drink*"[Title/Abstract] OR "caffeinated beverage*"[Title/Abstract] OR "pre-workout"[Title/Abstract] OR "pre workout"[Title/Abstract] OR "pre-workout supplement*"[Title/Abstract] OR "pre workout supplement*"[Title/Abstract] OR "multi-ingredient pre-workout supplement*"[Title/Abstract] OR "multi ingredient pre workout supplement*"[Title/Abstract] OR MIPS[Title/Abstract] OR guarana[Title/Abstract]) AND (addict*[Title/Abstract] OR dependen*[Title/Abstract] OR "caffeine dependence"[Title/Abstract] OR "caffeine addiction"[Title/Abstract] OR "caffeine use disorder"[Title/Abstract] OR "use disorder"[Title/Abstract] OR "problematic caffeine use"[Title/Abstract] OR "problematic use"[Title/Abstract] OR misuse[Title/Abstract] OR abuse[Title/Abstract] OR overuse[Title/Abstract] OR "excessive use"[Title/Abstract] OR "excessive intake"[Title/Abstract] OR "compulsive use"[Title/Abstract] OR craving[Title/Abstract] OR withdrawal[Title/Abstract] OR "caffeine withdrawal"[Title/Abstract] OR "withdrawal symptom*"[Title/Abstract] OR abstinence[Title/Abstract] OR cessation[Title/Abstract] OR discontinuation[Title/Abstract] OR tolerance[Title/Abstract] OR habituation[Title/Abstract] OR "habitual use"[Title/Abstract] OR "habitual caffeine use"[Title/Abstract] OR "regular caffeine use"[Title/Abstract] OR "chronic caffeine use"[Title/Abstract]) AND (athlet*[Title/Abstract] OR sport*[Title/Abstract] OR exercis*[Title/Abstract] OR "physical activity"[Title/Abstract] OR "physically active"[Title/Abstract] OR "trained individual*"[Title/Abstract] OR "trained participant*"[Title/Abstract] OR "recreationally active"[Title/Abstract] OR "recreational athlete*"[Title/Abstract] OR "competitive athlete*"[Title/Abstract] OR "elite athlete*"[Title/Abstract] OR "college athlete*"[Title/Abstract] OR "collegiate athlete*"[Title/Abstract] OR "student athlete*"[Title/Abstract] OR "university athlete*"[Title/Abstract] OR fitness[Title/Abstract] OR gym[Title/Abstract] OR "gym user*"[Title/Abstract] OR "fitness enthusiast*"[Title/Abstract] OR "resistance training"[Title/Abstract] OR "strength training"[Title/Abstract] OR "weight training"[Title/Abstract] OR bodybuilding[Title/Abstract] OR bodybuilder*[Title/Abstract] OR endurance[Title/Abstract] OR runner*[Title/Abstract] OR cyclist*[Title/Abstract] OR triathlete*[Title/Abstract])	15 June 2026
Web of Science Core Collection	TS=((caffeine OR caffeinated OR "caffeine supplement*" OR "caffeine supplementation" OR "caffeine intake" OR "caffeine consumption" OR "caffeine use" OR coffee OR "energy drink*" OR "caffeinated beverage*" OR "pre-workout" OR "pre workout" OR "pre-workout supplement*" OR "pre workout supplement*" OR "multi-ingredient pre-workout supplement*" OR "multi ingredient pre workout supplement*" OR MIPS OR guarana) AND (addict* OR dependen* OR "caffeine dependence" OR "caffeine addiction" OR "caffeine use disorder" OR "use disorder" OR "problematic caffeine use" OR "problematic use" OR misuse OR abuse OR overuse OR "excessive use" OR "excessive intake" OR "compulsive use" OR craving OR withdrawal OR "caffeine withdrawal" OR "withdrawal symptom*" OR abstinence OR cessation OR discontinuation OR tolerance OR habituation OR "habitual use" OR "habitual caffeine use" OR "regular caffeine use" OR "chronic caffeine use") AND (athlet* OR sport* OR exercis* OR "physical activity" OR "physically active" OR "trained individual*" OR "trained participant*" OR "recreationally active" OR "recreational athlete*" OR "competitive athlete*" OR "elite athlete*" OR "college athlete*" OR "collegiate athlete*" OR "student athlete*" OR "university athlete*" OR fitness OR gym OR "gym user*" OR "fitness enthusiast*" OR "resistance training" OR "strength training" OR "weight training" OR bodybuilding OR bodybuilder* OR endurance OR runner* OR cyclist* OR triathlete*))	15 June 2026
Scopus	TITLE-ABS-KEY((caffeine OR caffeinated OR "caffeine supplement*" OR "caffeine supplementation" OR "caffeine intake" OR "caffeine consumption" OR "caffeine use" OR coffee OR "energy drink*" OR "caffeinated beverage*" OR "pre-workout" OR "pre workout" OR "pre-workout supplement*" OR "pre workout supplement*" OR "multi-ingredient pre-workout supplement*" OR "multi ingredient pre workout supplement*" OR MIPS OR guarana) AND (addict* OR dependen* OR "caffeine dependence" OR "caffeine addiction" OR "caffeine use disorder" OR "use disorder" OR "problematic caffeine use" OR "problematic use" OR misuse OR abuse OR overuse OR "excessive use" OR "excessive intake" OR "compulsive use" OR craving OR withdrawal OR "caffeine withdrawal" OR "withdrawal symptom*" OR abstinence OR cessation OR discontinuation OR tolerance OR habituation OR "habitual use" OR "habitual caffeine use" OR "regular caffeine use" OR "chronic caffeine use") AND (athlet* OR sport* OR exercis* OR "physical activity" OR "physically active" OR "trained individual*" OR "trained participant*" OR "recreationally active" OR "recreational athlete*" OR "competitive athlete*" OR "elite athlete*" OR "college athlete*" OR "collegiate athlete*" OR "student athlete*" OR "university athlete*" OR fitness OR gym OR "gym user*" OR "fitness enthusiast*" OR "resistance training" OR "strength training" OR "weight training" OR bodybuilding OR bodybuilder* OR endurance OR runner* OR cyclist* OR triathlete*))	15 June 2026

Notes: No publication-date restriction was applied beyond the final search date. Searches were limited to the stated bibliographic fields; reference-list checking and forward citation tracking were conducted separately as described in the Methods.
